# Cellulose hydrolysis using ionic liquids and inorganic acids under dilute conditions: morphological comparison of nanocellulose†‡

**DOI:** 10.1039/d0ra05976e

**Published:** 2020-10-28

**Authors:** Jacobs H. Jordan, Michael W. Easson, Brian D. Condon

**Affiliations:** USDA, Agricultural Research Service, Southern Regional Research Center 1100 Robert E. Lee Blvd New Orleans LA 70124 USA michael.easson@usda.gov

## Abstract

The preparation of cellulose nanocrystals (CNCs) from cellulose extracted from cotton gin motes (CGM) using an ionic liquid (1-butyl-3-methylimidazolium chloride, [BMIm]Cl) under dilute conditions is reported. The concurrent process involves minimal swelling of cellulose with an ionic liquid and hydrolysis of the cellulose initiated by the addition of either phosphoric (H_3_PO_4_), hydrochloric (HCl), or sulfuric (H_2_SO_4_) acid. The obtained nanocrystals had similar physical properties (*e.g.* crystallinity) to the counterparts prepared under conventional conditions and exhibited superior thermal properties for sulfate CNCs. Additionally, the obtained CNCs had low surface functionalization, yet were colloidally stable for >90 days, which is a desirable trait for post-functionalization of CNCs. This process represents a general strategy utilizing dilute ionic liquids in the preparation of nanocellulose under mildly acidic conditions.

## Abstract 2

Cellulose nanocrystals prepared with 1-butyl-3-methylimidazolium chloride ([BMIm]Cl) and HCl, H_2_SO_4_, or H_3_PO_4_ in a one-pot process under dilute conditions are investigated for changes in CNC dimensions, crystallinity, and thermal stability.

## Graphical abstract

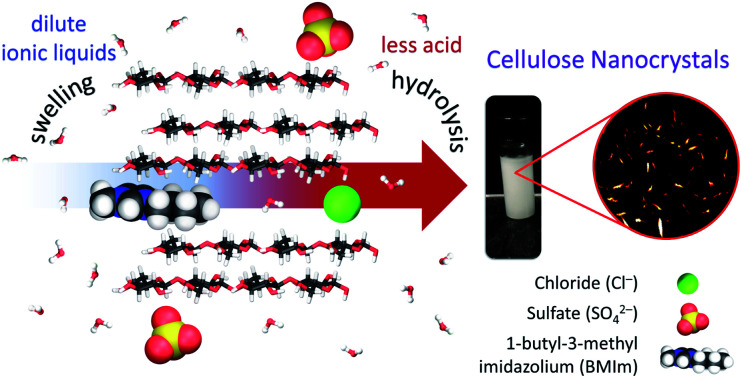

## Introduction

Ionic liquids (ILs) are regarded as a “green solvent” and possess favourable properties; they have a low melting point (<100 °C), are recoverable, and are readily tailored to specific applications.^1,2^ ILs have increasingly been applied in processing lignocellulose biomass.^3–6^ For example, an IL (1-butyl-3-methylimidizolium chloride, [BMIm]Cl) was used to dissolve cellulose with the aid of a solid acid support.^5^ In another example, Xia *et al.*^7^ screened a number of ionic liquids and deep eutectic solvents (DES) for suspension pre-treatment of cellulose biomass. The rate of enzymatic hydrolysis was affected using neat, 1.0, or 2.0 M ILs, and aqueous ILs (1.0 or 2.0 M) were found to be an effective suspension pre-treatment.

The advent of IL use in biomass processing has more recently increased with the use of acidic ionic liquids.^8^ Thus, ILs have also been utilized for the hydrolysis of cellulose to produce cellulose nanocrystals (CNCs) and nanofibers (CNFs) from various lignocellulose sources.^9^ This is in contrast to traditional methods to prepare CNCs from the hydrolysis of cellulose using strong minerals acids: hydrochloric acid (HCl), sulfuric acid (H_2_SO_4_), and phosphoric acid (H_3_PO_4_).^10–13^ Mao *et al.* described the use of mildly acidic ionic liquids for the production of CNCs; they compared the case of the neat protic ionic liquid (PIL) obtained from BMIm and hydrogen sulfate ([BMIm]HSO_4_) and dilute IL premixed with H_2_O (1 : 14.3, mol mol^−1^).^14^ Yields were considerably improved with the use of the dilute IL [BMIm]HSO_4_ (*ca.* 48 *vs.* 90%, respectively). In contrast, Yang *et al.* used neat ILs as a pretreatment for the dissolution and regeneration of cellulose prior to H_2_SO_4_ hydrolysis or TEMPO oxidation; the resulting CNCs were obtained in low yield and had very low (<25%) crystallinity.^15^

Lazko *et al.* prepared thermally stable CNCs *via* a two-step swelling/hydrolysis method using the neat ionic liquid [BMIm]Cl for 1 h follow by hydrolysis for 2, 5 or 16 h upon the addition of H_2_SO_4_ (4, 2 or 1 wt%, respectively).^16^ This method was improved with a Brønsted acid IL 1-(4-sulfobutyl)-3-methylimidazolium hydrogen sulfate ([SBMIm]HSO_4_) that was effectively used for the production of CNCs. Firstly, cellulose fibres were swollen in anhydrous [BMIm]Cl and then hydrolysed by the subsequent addition of [SBMIm]HSO_4_ at rather low concentrations (<4%).^17^ A more recent alternative used a combination of swelling in [BMIm]Cl (45 °C, 2 h) followed by 2-fold addition of oxalic acid (70 wt%) to initiate hydrolysis; optimum conditions were 7 h and 90 °C.^18^

In another two-step procedure,^19^ cellulose from bleached hard and softwood pulps and microcrystalline cellulose (MCC) from cotton linters were swollen in neat [BMIm]HSO_4_ for 24 h and then diluted with water to catalyse the formation of CNCs; reaction conditions were 12 h, ≥100 °C, and H_2_O : IL ratio of 1 : 4. This resulted in CNCs with low sulfur content and high crystallinity (77–82%).^19^ Treatment of microcrystalline cellulose (MCC) from cotton linters with neat [BMIm]HSO_4_ for 1.5 at *T* ≥ 90 °C similarly resulted in highly crystalline (>90%) CNCs.^20^

Iskak *et al.* investigated the effect of hydrolysis temperature and time on CNC production using neat [BMIm]Cl;^21^ they showed increasing temperature and time resulted in more crystalline CNCs with higher CNC yield and improved aspect ratio. In another instance, an ultrasonic pre-treatment in combination with an IL was employed to allow extraction of CNCs using milder acid hydrolysis (20–23 wt% H_2_SO_4_); the sulfuric acid was added directly to the suspension in a 2-step, tandem, 1-pot process.^22^ CNCs with improved thermal stability and re-dispersibility were prepared by using the ionic liquid [BMIm]BF_4_ by a simple rotary evaporator procedure.^23^ The PIL [BMIm]HSO_4_ was used to prepare CNCs (80 °C, 3 h); esterified CNCs with long chain fatty acids from methyl laurate were also prepared using a binary mixture of [BMIm]HSO_4_ and [BMIm]BF_4_ in conjunction with a lipase *via* a lipase-catalysed transesterification.^24^

In addition to the preparation of nanocellulose, ILs have been used as a solvent and reagent for preparation of functionalized CNCs. Tetrabutylammonium acetate (TBAA) was shown to dissolve cellulose in the presence of dimethylsulfoxide (DMSO) or crown ethers without pretreatment;^25^ subsequently TBAA used in combination with DMSO or *N*,*N*-dimethylacetamide (DMA) and acetic anhydride to produce hydrophobic, acetylated CNCs in a one-pot process, which were added as a support in a PLA matrix.^26^ Partially acetylated CNCs were prepared from successive rounds of pulping/processing of extractive free wood with the IL 1-ethyl-3-methylimidazolium acetate [EMIm]OAc at 60 °C for 2 h.^27^

Different extraction protocols lead to differing properties such as length, crystallinity, aspect ratio, and amount and type of surface functional groups. For example, hydrolysis with HCl gave hydroxyl CNCs that were selected as a toughening agent in epoxy adhesives^28^ and a two-step oxidation/reduction strategy afforded sterically stabilized neutral CNC suspensions.^29^ In contrast, the use of [BMIm]Cl and oxalic acid promoted the formation of CNCs with the carboxylate moiety; the CNCs were characterized with high crystallinity (80%) and both longer (>300 nm) and wider (∼37 nm) than H_2_SO_4_ hydrolysed CNCs.^18^ The production of nanocellulose with acid blends resulted in different morphologies and allowed fine-tuning of the number of surface charge groups and zeta (*ζ*) potential.^11^ Sulfate-modified CNCs were used to stabilised oil-in-water Pickering emulsions, and it was found that the amount of CNCs required for stabilisation was reduced for CNC suspension with a lowered *ζ*-potential (*i.e.* with a lower surface charge density),^30^ and similarly, sulfate CNCs were modified with an amino terminated polystyrene to render amphiphilic CNCs for enhanced emulsion stabilisation.^31^ CNCs derived from cotton had a greater aspect ratio compared to wood CNCs, which was found to have a beneficial impact on the toughening effect of polyvinyl alcohol composites.^32^

Other examples have sought to reduce the costs associated with the use of the (usually recoverable) IL. For example, a custom PIL was prepared by neutralization of ethanolamine and sulfuric acid and was used to prepare CNCs; however, the CNCs were characterized with low *ζ* potential and crystallinity (−19.1 mV and 54%, respectively).^33^ Additionally, neat imidazole or water-imidazole mixtures (25% w/w) were used to prepare CNFs or CNCs, respectively, after heating at 120 °C for 24 h.^34^

In this work and inspired by previously reported results, a strategy employing the IL [BMIm]Cl, coupled with three different inorganic acids under dilute conditions was investigated for the hydrolysis of cellulose to produce CNCs. As a benchmark comparison, CNCs were prepared by mineral acid hydrolysis under conventional conditions and the physicochemical properties compared to those obtained using dilute acids and ILs. The results are presented and discussed herein.

## Experimental

### Materials

#### Raw materials

Cotton gin motes (CGM) were obtained from the USDA Research Facility in Stoneville, Mississippi. Chemicals and supplies were purchased from either Millipore-Sigma Corporation or VWR-USA and were used as received. All water sources used deionized water with a maximal conductivity of ≤1.0 μS cm^−1^.

#### Extraction of cellulose

The CGM were mechanically ground to 20 mesh with a Wiley mill (E3300, Eberbach Corp.). Celluloses were extracted by subsequent alkali and bleaching treatments (Scheme S1‡).^13^ For the alkali treatment, a 4% solution of sodium hydroxide (NaOH) was used for 2 h at 70 °C followed by exhaustive washing of the fibres with water until the eluent was near neutral (pH ≈ 6–7). For the bleaching treatment, an acidified sodium chlorite (NaClO_2_) solution (1.0% acetic acid (v/v) and 0.50% NaClO_2_ (w/v)) was used for 2 h at 75 °C; bleaching was repeated 2–3 times until the fibres were fully white. In both instances, the fibre to liquor ratio was 1 : 20 (w/v). The recovered cellulose fibres were washed thoroughly with water until a colourless eluant was obtained and then oven-dried (70 °C) to a constant mass.

#### Characterization of cellulose

The content of cellulose, hemicellulose and lignin of the extracted cellulose were determined using a standard fibre analysis method for chemical analysis of biomass samples for structural carbohydrates, and lignin.^35^ The sample composition was corrected for moisture content. Water/ethanol extracted samples were treated using a two-stage H_2_SO_4_ hydrolysis method to convert structural carbohydrates into monomeric sugars. Refined sugar controls were run in parallel to correct for losses. Acid insoluble solids were washed, dried, and weighed to determine acid insoluble lignin. Sugars and organic acids were analysed using an Ultimate 3000 HPLC (Thermo Fisher Scientific) equipped with a refractive index detector. Eluents were separated using separate injections on organic acid (Aminex HPX-87H column, Bio Rad Laboratories) and sugar (Aminex HPX-87P column, Bio Rad Laboratories) columns operated according to the manufacturers' specifications.

### Nanocrystal preparation

#### Nanocrystals from mineral acid hydrolysis

H_3_PO_4_, H_2_SO_4_, and HCl-hydrolysed CNCs, respectively pCNC, sCNC, and hCNC, were prepared from cellulose fibres extracted from CGM by minor modifications to the procedures supplied previously.^10,12,13^ H_2_SO_4_ hydrolysis was conducted for 60 min at 60 °C using 62% (w/w) H_2_SO_4_ at a material to liquid ratio of 1 : 20 (w/v). The resulting suspension was quenched by dilution four-fold with ice water and then washed until pH ≥ 5 by successive centrifugation cycles at 16 000 × *g* for 15 min each cycle. The crystals (sCNC) were then dispersed using a 750 W ultrasonic processor (Vibra-Cell probe sonicator, VCX-750, Sonics & Materials) with a power amplitude of 60% (20 kHz frequency) for 5 min. The obtained suspension was centrifuged at 3500 × *g* for 5 min (to sediment larger particulates and contaminates), filtered under vacuum with a Whatman glass microfiber filter (grade GF/F, 0.7 μm) and dialyzed using a regenerated cellulose dialysis tubing (MW cut-off 10 000) for several days until the solution conductivity stabilized for two successive bath changes (measured value < 2 μS cm^−1^). Hydrolysis with HCl (hCNC) and H_3_PO_4_ (pCNC) were similarly conducted using 12% and 74% (w/w) HCl or H_3_PO_4_ at 100 °C for 120 and 90 min, respectively, using 75 or 125 mL of the acid solution per gram of cellulose. Work-up and processing conditions were as described for H_2_SO_4_ hydrolysis. The suspensions were subsequently stored in a sealed plastic container and refrigerated (4–8 °C) between uses. The yield and concentration of the nanocrystals was determined gravimetrically.

#### Nanocrystals from dilute ionic liquids and acids

The ionic liquid [BMIm]Cl was used in all cases mixed with deionized water at a molar ratio of 1 : 33. The mixture (13.3 g) was added to the extracted cellulose (0.75 g) and stirred for 10 min. After the cellulose fibres were fully suspended the inorganic acid was added for a molar ratio of 1 : 1 : 33. (IL : acid : H_2_O) and the suspension heated to 75 °C for 20 h (or 90 °C, 44 h in the case of H_3_PO_4_). Each hydrolysis was then quenched by dilution to a total volume of ∼40 mL with iced water and then centrifuged 12 000 × *g* for 15 min. The IL-containing supernatant was decanted, and the CNC-containing precipitate was repeatedly washed until a turbid supernatant was obtained. Between each wash, ultrasonication was used to re-suspend the precipitate. The obtained suspensions of CNCs from IL-mediated hydrolysis using HCl (hCNCi), H_2_SO_4_ (sCNCi), and H_3_PO_4_ (pCNCi), collectively denoted IL-CNCs, were dialyzed and stored at 4 °C. The yield and concentration of the nanocrystals was determined gravimetrically.

### Characterization

#### Atomic force microscopy

The atomic force microscopy (AFM) measurements were performed with an Agilent 500 atomic force microscope. Data was collected in contact mode using a triangular shaped Pyrex nitride cantilever with a gold reflex coating and silicon nitride tips (0.32 N m^−1^ force constant, 67 kHz resonance frequency, NanoWorld). For determination of the CNC length and diameter (height), mica discs (V1 AFM Mica Discs, 20 mm, TedPella, Inc.) were pre-treated with 100 μL of a poly-l-lysine solution (0.01 wt%) and rinsed thoroughly with water for two minutes and then blown dry with a stream of argon. CNC suspensions (0.01 wt%) were applied by the drop cast method and rinsed after two minutes and blown dry. AFM height measurements were determined by use of the section analysis tool provided with the AFM software (picoview 1.14) on a 4 × 4 μm image from at least 100 individual observations. ImageJ software v. 1.52d was used for analysis of the particle length.^36^ Results were fit with a Gaussian function using OriginPro 2018b (OriginLab Corporation, Northampton, MA) to determine the mean length and height of the CNCs.

#### Conductometric titrations

The content of the surface acid groups (and hence sulfur (S) or phosphorus (P) content) on CNCs was measured by conductometric titrations as described previously with minor modifications.^37–40^ The titrations were performed on CNCs that were converted to their acid from by chromatography over a large excess of Dowex® Marathon™ C Hydrogen from ion-exchange resin (23–27 mesh). CNC suspensions, 5–10 mL, 0.5–1.0 wt%, were diluted to 100 mL in 100 μM sodium chloride (NaCl). Throughout each titration, the conductivity was continuously monitored, and 100 μL aliquots of standardized 2.0 mM NaOH was added over 45–60 min. The volume-corrected conductivity was plotted, and the equivalence point was determined by the intersection of least-squares regressions from the positive and negative sloped regions. Data was collected in (at least) triplicate for each sample. The surface charge density in e nm^−2^ can then be calculated from the content of S or P determined from the conductometric titrations. The surface charge density *σ* is determined from the total mole equivalents (mol) of S or P in 1 g of CNCs by eqn (1):^41^1
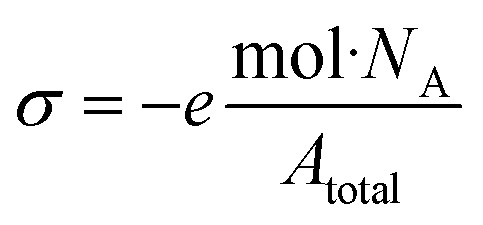
where *e* is the elementary charge, *N*_A_ is Avogadro's number, and *A*_total_ is the surface area of the CNCs assuming a cylindrically shaped rod, (see ESI‡ for details).

#### Dynamic light scattering (DLS)

CNC suspensions were analysed for particle size (hydrodynamic radius, *r*_H_) and distribution using a Malvern Zetasizer Nano. Dilute suspensions (0.01 wt%) were sonicated for 10 min in an ultrasonic bath (Cole-Parmer, 42 kHz ultrasound frequency, 100 W power), centrifuged (6000 × *g*, 10 min), and filtered through a 0.45 μm PVDF filter to remove larger particulates and dust.^42,43^ In each instance at least 10 measurements were recorded and the data acquired in triplicate for separate samples. The results were averaged and plotted using the Malvern software for the number-weighted size distribution.

#### *ζ*-Potential

The CNC suspensions were diluted to 0.20 wt% and filtered through a 0.45 μm PDVF filter. The suspensions were analysed in a folded capillary cell for electrophoretic mobility and hence the *ζ*-potential using a Malvern Zetasizer Nano. *ζ*-Potential measurements were collected with 1 mM NaCl buffer. The *ζ*-potential of CNC samples was measured using the Smoluchowski approximation of Henry's function for aqueous dispersions with a suitable electrolyte concentration, such that the electric double-layer thickness around the CNCs is thin compared to the particle size. CNC dispersions were measured in triplicate at 25 °C with each measurement consisting of at least 15 cycles. The error is presented as the standard deviation from individual measurements.

#### X-ray photoelectron spectroscopy (XPS)

The elemental composition of CNCs was measured using XPS on lyophilized powders of CNCs. The XPS measurements were conducted on a VG Scientific MKII system using an Al Kα anode as excitation source (*hν* = 1486.6 eV). The pressure in the chamber during analysis was <2 × 10^−8^ mbar. For the survey scan, data acquisition was done with dwell time of 6 s. To improve the signal to noise ratio 60 s dwell time was used for C 1s (275–295) and O 1s (522–542) regions. For acquisition of the S 2p (157–177) and P 2p (123–143) regions, a 200 s dwell time was used. After data acquisition, the signal of each data point was scaled to a normalized dwell time of 60 seconds. The spectra were calibrated by referencing the C 1s peak at 284.8 eV binding energy. From the determined mass concentrations, the number (*n*) of sulfate or phosphate groups per 100 bulk anhydroglucose units was calculated based on eqn (2) and (3):^44^2
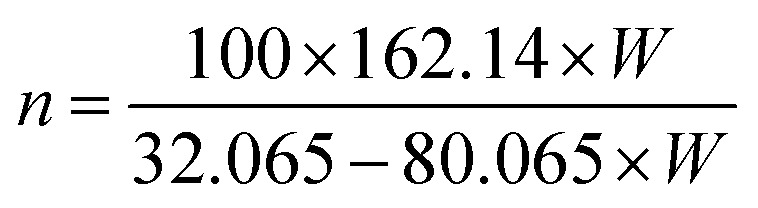
3
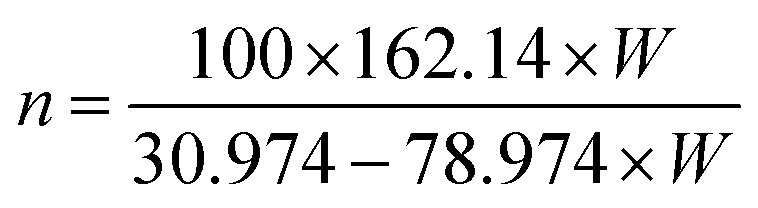
where *W* (wt%) is the mass concentration of sulfur or phosphorous element in samples of CNCs.

#### Thermogravimetric analysis (TGA)

Thermal analysis of lyophilized CNCs was acquired with a TA Instruments Q500 thermal analyser. The corresponding first-order derivatives of the thermograms (DTG) reveal the temperature at the maximum rate of weight loss. Samples (2–4 mg) were analysed in platinum crucibles under a nitrogen atmosphere with a 10 mL min^−1^ sample gas flow rate, heating rate of 10 °C min^−1^, and temperature range from 30 °C to 650 °C. Data was analysed with the aid of the Universal Analysis 2000 software package (v. 4.5, TA Instruments – Waters, LLC, New Castle, DE) and plotted using OriginPro 2018b.

#### Powder X-ray diffraction (XRD)

XRD measurements were performed with a PANalytical Empyrean laboratory diffractometer (Malvern Panalytical Inc., Westborough, MA, USA) using Cu Kα-radiation (1.5406 Å) with a spinning, zero-background sample holder at room temperature and a PIXcel3D detector equipped with a 1.0 mm radial divergence slit and a 0.1 mm receiving slit. Powder diffraction patterns were analysed with a pseudo-Voigt peak shape using the MAUD Rietveld refinement program (Materials Analysis Using Diffraction, v. 2.84). Crystallographic information files for cellulose Iβ and cellulose II were used for the crystalline and amorphous phases respectively.^45,46^ For the amorphous content the isotropic crystallite size was set to twelve. Crystallinity index (CrI) was calculated using the Rietveld analytical method and is calculated as the area of the calculated pattern for crystalline cellulose (*A*_c_) after background subtraction over the sum of the total contributions from both the crystalline and amorphous (*A*_a_) phases (eqn (4)).^47^4
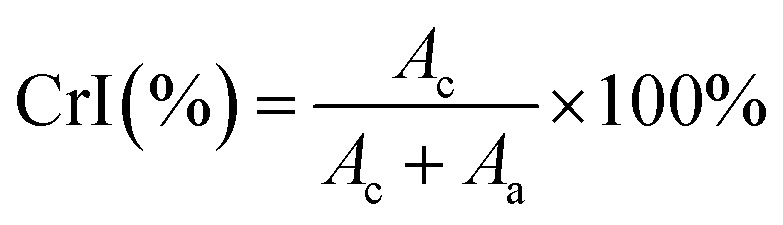


The Scherrer equation (eqn (5)) was used to calculate (a measure) of the crystallite size, *L* (nm), perpendicular to the (*hkl*) plane:^48^5
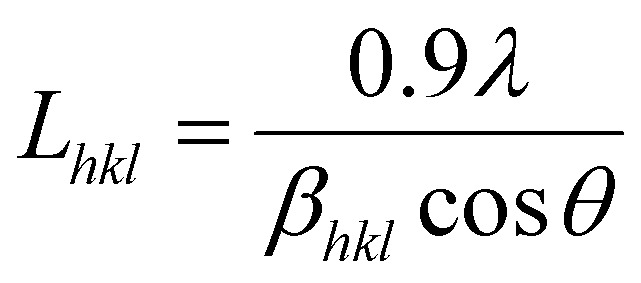
where, *λ* is the radiation wavelength (1.5406 Å), *θ* is the diffraction angle, and *β*_*hkl*_ is the angular full-width at half the maximal height (FWHM), in radians, of the respective line profile. The associated *d*-spacings were calculated from the refined unit cell dimensions.

## Results and discussion

### Extraction of cellulose

**Fig. 1:**
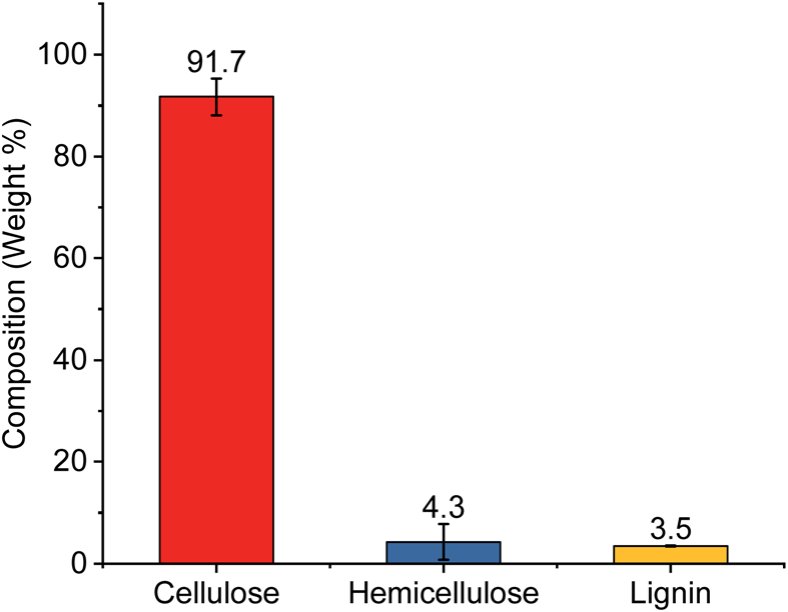
Composition of extracted celluloses used to prepare CNCs.

Celluloses were extracted using subsequent alkali and bleaching treatments from cotton gin motes (CGM). The starting cotton gin motes were previously shown to contain roughly 67% celluloses and the remaining amount of the material is comprised of lignin (13.5%), and extractables (9.6%), as well as hemicelluloses and other sugar polymers (7.5%).^13^ Following chemical processing the extracted cellulose powder contained >90% cellulose with small amounts of hemicellulose and lignin (Fig. 1). This represents roughly a 50% increase in the amount of cellulose available for conversion to various CNCs, complete removal of the extractables, and a substantial reduction in the amount of hemicellulose (∼43%) and lignin (∼75%) present in the source material.

### Preparation of cellulose nanocrystals

The results of cellulose hydrolysis indicate that roughly one-third of the cellulose was hydrolysed into its constituent sugars during the IL-dilute acid treatment. Additionally, the combination of IL and the inorganic acids generated nanocellulose. Under dilute conditions the combination rendered both fully hydrolysed monosaccharides and incomplete hydrolysis, leading to microcrystalline cellulose. However, these components are readily separated by centrifugation. This is comparable to the results obtained using strong mineral acids. Subsequent work-up to remove the non-hydrolysed cellulosic solid residues (CSR) from the CNC fractions gave slightly lower yields of the obtained CNC suspension in all cases (Table 1). However, these yields are on par with those reported in the literature,^10–12^ and CSR may be subsequently converted to CNFs.^4,49,50^ It is apposite to note that many reported conventional work-ups indicated a total yield of isolated non-hydrolysed cellulose without adequately separating CSR from the CNCs, resulting in CNC suspensions that still contain contaminates of MCC and thus leading to substantial opacity at relatively lower concentrations (<1 wt%).

**Table 1:** Conditions (time, temperature, and acid equivalents) and results (soluble sugars, CNC yield, and cellulosic solid residues) obtained using traditional and ionic-liquid mediated hydrolysis of cellulose^a^

Entry	Sample	Time (h)	Temp (°C)	Acid ratio (equiv. per unit^b^)	Sol. sugars^c^ (%)	Yield^d^ (%)	CSR^e^ (%)
1	hCNC	1.5	100	48	10	63	27
2	sCNC	1	60	29	44	55	<1
3	pCNC	2	100	230	22	67	11
4	hCNCi	20	75	3.5	38	53	9
5	sCNCi	20	75	3.5	34	40	26
6	pCNCi	44	90	3.5	36	43	21

a CNCs were prepared with either HCl (hCNC), H_2_SO_4_ (sCNC), or H_3_PO_4_ (pCNC) as the acid source, while the “i” suffix (*e.g.* hCNCi, sCNCi, or pCNCi) indicates use of the ionic liquid (1-butyl-3-methylimidizolium chloride) during the preparation.

b Per anhydroglucose unit of cellulose.

c Soluble sugars total weight were determined from the difference in the mass of total cellulosic residues before and after hydrolysis prior to separation of the CNCs and non-hydrolysable cellulosic solid residues (CSR).

d CNC yields were determined gravimetrically after separation of the CSR from the suspension.

e CSR yields were determined from the total isolated mass of residual cellulosic solids after exhaustive centrifugation and sonication.

Hydrolysis with the IL and HCl for 20 h at 75 °C gave CNCs in modest yield (53%) and low residual CSR (9%). Identical conditions were less effective for H_3_PO_4_ hydrolysis giving a low 12% yield and substantial amount of CSR (57%). This was not surprising given that typically hydrolysis using concentrated (74%) H_3_PO_4_ requires significantly harsher reaction conditions than those that were employed using IL-mediated hydrolysis. The reaction time for H_3_PO_4_ hydrolysis was thus increased in the presence of the IL to 44 h and the reaction temperature to 90 °C, which greatly improved the yield (43%) and reduced the CSR to 21% during hydrolysis, with a concomitant increase in the surface functionalisation. For comparison, traditional hydrolysis conditions using 4 M HCl (∼12% w/w) or 11.55 M H_3_PO_4_ (74% w/w) were conducted using approximately 48 or 230 equiv. of the acid per anhydroglucose unit of cellulose, whilst hydrolysis using the more dilute acids employed with the IL used only 3.5 equiv. of the acid. This corresponds to a reduction of 93% and 98% in the amount of acid used on a stoichiometric basis. Additionally, milder temperatures (75–90 °C) were employed compared to 100 °C for traditional hydrolysis conditions.

As a possible explanation, Xia *et al.*^7^ showed that suspension pretreatment of cellulose with 1.0 M BMIm was more effective when “cellulose dissolving” counterions such as acetate (AcO^−^), and chloride (Cl^−^) were used. These anions are strongly solvated (Δ*G*_hdr_ = −373 and −347 kJ mol^−1^, respectively)^51^ suggesting solvation of the anion enhances the efficacy of the IL counterion. For the example containing H_2_SO_4_, when H_2_SO_4_ is added, the first ionisation (p*K*_a_ = −3) gives HSO_4_^−^ (Δ*G*_hdr_ = −335 kJ mol^−1^). Collins law of matching water affinities^52,53^ suggests a greater propensity for HSO_4_^−^ to form a contact or solvent separated ion pair with BMIm, thereby reducing its effectiveness compared to HCl and Cl^−^. In the case of H_2_PO_4_^−^ (Δ*G*_hdr_ = −473 kJ mol^−1^), incomplete ionisation (first p*K*_a_ = 2.16) suggests despite the greater solvation of H_2_PO_4_^−^ there is less available acid to initiate glycosidic bond cleavage, and hence harsher conditions were required.

Overall conditions were thus less improved for H_2_SO_4_ hydrolysis of cellulose. Literature yields for H_2_SO_4_ hydrolysis typically vary between 20–70% for hydrolysis of soft or hardwood pulps and cellulose from cotton depending on the method of isolation. Under the employed conditions, the IL-mediated hydrolysis gave a slightly lower yield and a significant amount of CSR compared to concentrated acid hydrolysis, providing comparable amounts of nanocellulose, monosaccharides and MCC.^16^ It is worth noting, the amount of acid was reduced eight-fold, albeit at the expense of longer reaction time and elevated temperature. Thus, while each treatment successfully obtained CNCs, the use of IL-mediated hydrolysis with HCl and H_3_PO_4_ proved more economical (in terms of acid consumption and waste products) than isolation of CNCs using concentrated acids. The use of H_2_SO_4_ under similar conditions proved a less effective alternative, in agreement with similar work.^16^

### Atomic force microscopy

**Fig. 2:**
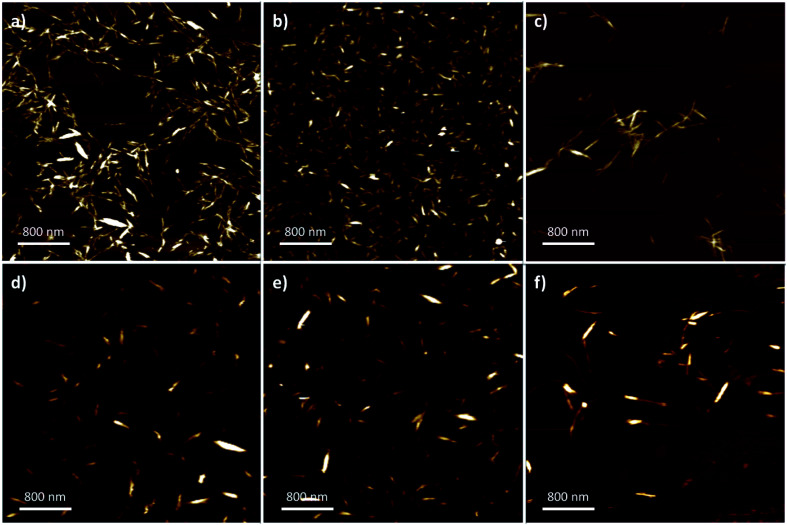
Representative 4 × 4 AFM images from: (a) hCNC; (b) sCNC; (c) pCNC; (d) hCNCi; (e) sCNCi, and; (f) pCNCi.

Nanoparticle dimensions and aspect ratio affect macroscopic properties in end-use applications such as composite fibers,^32^ reinforcements,^28,54^ fillers,^55^ stabilisers,^56^ and film assembly.^57,58^ To characterize the nanocrystal dimensions, AFM (Fig. 2) was used to analyse the nanoparticle length, width, and aspect ratio (Table 2). The nanoparticle width was determined with the aid of the section analysis toolkit provided with the AFM software, and ImageJ was used for analysis of the nanoparticle length (ESI: Fig. S2 and S3‡).^36^ The standard conditions using strong mineral acids promoted a narrower size-distribution and smaller observed crystalline sizes with the majority having lengths of 100–400 nm and widths of 5–15 nm. Specifically, sCNC were obtained with the shortest length, width, and smallest aspect ratio, while differences in the dimensions of hCNC and pCNC were not considered significant.

**Table 2:** Physical properties of CNCs determined by atomic force microscopy

Entry	Sample	Length^a^ (nm)	Height^b^ (nm)	Aspect ratio^c^
1	hCNC	218 ± 69	10.4 ± 3.8	21.0
2	sCNC	102 ± 51	7.2 ± 2.2	14.2
3	pCNC	216 ± 77	7.5 ± 3.5	28.8
4	hCNCi	223 ± 60	10.3 ± 3.7	21.7
5	sCNCi	198 ± 85	9.7 ± 3.7	20.4
6	pCNCi	322 ± 149	11.4 ± 3.0	28.2

a Determined using section analysis software on the AFM instrument.

b Determined using ImageJ software.

c Determined from the ratio of the mean sample length to the mean sample height.

In contrast, the IL-hydrolysis conditions gave slightly longer nanocrystals in all cases, typically 200–300 nm with some individual crystals up to 800 nm. There was no significant difference observed among IL-CNCs, although the dimensions of sCNCi were comparably longer than sCNC. Generally, the AFM height, or width of the IL-CNCs was marginally larger (approx. 10 nm). In the case of both pCNC and pCNCi a significantly greater aspect ratio was observed (∼28). However, aspect ratios were comparable to literature precedent for those produced by conventional methods. The aspect ratio of CNCs is correlated with an increased elastic modulus and tensile strength, and affects the reinforcement capabilities of CNC-polymer nanocomposites.^32,59–61^ In film applications, longer CNCs with a greater aspect ratio give more porous surfaces than shorter CNCs with a lower aspect ratio, which provide for more complete surface coverage.^62^ This directly affects their application in gas adsorption and separations.^63^ Thus, proper selection of processing conditions is crucial with end-use applications of CNCs in mind.

### Conductometry

Hydrolysis with H_3_PO_4_ and H_2_SO_4_ gives CNCs with phosphate and sulfate half-esters, –OPO_3_^2−^ and –OSO_3_^−^ respectively, while hydrolysis with HCl gives CNCs with the hydroxyl (–OH) moiety. Typical phosphate content is <40 mmol kg^−1^, while sulfate content varies from 80–300 mmol kg^−1^.^12,62^ Given the high sulfate content that can be expected from traditional hydrolysis conditions, it can be more convenient to achieve colloidally stable CNCs with low surface functionality during synthesis rather than by incorporating a post-synthetic strategy to hydrolyse the surface sulfate groups.^40,64^ It can be expected, given the significantly reduced concentration of acid employed, that the obtained CNCs from IL-mediated hydrolysis would have low surface functionality, which is beneficial when considering post-functionalization or specific applications. For example, the concentration of –OSO_3_^−^ is correlated with CNC self-assembly^65^ and inversely related to CNC thermal stability.^10^ This limits their use in, for example, melt extrusions;^66^ moreover, –OPO_3_^2−^ or –OSO_3_^−^ functional groups impede post-synthetic modification of CNCs.^67^

CNC samples were analysed by conductometry to compare the CNCs prepared under conventional conditions (hCNC, sCNC, and pCNC) and those prepared by IL-mediated hydrolysis (hCNCi, sCNCi, and pCNCi). It should be noted that hCNC and hCNCi bear terminal hydroxyls and are only weakly acidic, thus conductometry was not useful in their characterization. Conductometric titrations (Fig. S1‡) were performed to measure the number of strong acid groups and hence the amount of –OPO_3_^2−^ or –OSO_3_^−^ present, using procedures reported previously.^37–40^ The number of –OPO_3_^2−^ or –OSO_3_^−^ groups detected by the consumption of available protons can then be readily converted to the P or S content (mmol P or S per kg of CNC) at the CNC surface (Table 3). This method does not generally detect P or S that are not acidic or the second proton of the acidic –OPO_3_^2−^ moiety.^12^ (see ESI‡ for details).

**Table 3:** Results of conductometry, light scattering and X-ray photoelectron spectroscopy

Entry	Sample	Conductometric titrations			Light scattering		X-ray photoelectron spectroscopy	
		S or P content (mmol kg^−1^)	S^a^ or P^a^ (%)	*σ* c (e nm^−2^)	*r*_H_ d (nm)	*ζ*-Potential (mV)	S^f^ or P^f^ (%)	Acidic groups per 100 glucose units (*n*)^g^
1	hCNC	—b	—b	—b	82 ± 24	−15.7 ± 0.9 e	—b	—b
2	sCNC	260 ± 6	0.83	−0.436 ± 0.010	21 ± 8	−42.8 ± 1.3	0.84	4.3
3	pCNC	36 ± 1	0.11	−0.064 ± 0.002	63 ± 16	−20.2 ± 1.4	0.25	1.3
4	hCNCi	—b	—b	—b	75 ± 20	−10.2 ± 4.1^e^	—b	—b
5	sCNCi	20 ± 7	0.06	−0.046 ± 0.016	75 ± 12	−24.2 ± 1.7	0.20	1.0
6	pCNCi	34 ± 4	0.11	−0.092 ± 0.011	64 ± 10	−25.0 ± 2.2	0.22	1.1

a Determined from S or P content from conductometric titrations.

b The hydrolysis conditions do not impart acidic sulfate or phosphate groups onto the CNC surface.

c Calculated from moles of S or P in 1 gram of CNCs assuming a cylindrical shape.

d The *r*_H_ is the apparent hydrodynamic radius obtained from DLS.

e Samples flocculated and reliable *ζ*-potential could not be obtained.

f Elemental mass% calculated from atom% from XPS.

g Determined from elemental mass% calculated from XPS.

Hydrolysis with 62% H_2_SO_4_ gave sCNC with 260 ± 6 mmol kg^−1^ –OSO_3_^−^, which is on par with literature precedent.^62^ IL-mediated hydrolysis with H_2_SO_4_, gave sCNCi that contained an order of magnitude less –OSO_3_^−^ (20 ± 7 mmol kg^−1^). In contrast, hydrolysis with H_3_PO_4_ or IL-mediated hydrolysis with H_3_PO_4_ gave a roughly comparable typical concentration of –OPO_3_^2−^ (34 ± 1, and 36 ± 4, respectively).^10,12^

The values for S or P content were used to calculate the surface charge density of the CNCs using eqn (1), and were used to converted to %S or %P for comparison to the values determined by XPS (*vide infra*). While the ratio of S found in sCNC compared to sCNCi is 13 : 1, due to the larger size of the sCNCi and hence smaller surface area per unit volume, the ratio of the surface charge density is reduced (∼9.5 : 1). The situation is reversed for pCNC and pCNCi, where the ratio of the content of P is approximately 1 : 1, the surface charge density is 1 : 1.4 with pCNCi having the larger value of surface charge density.

### Light-scattering

#### Dynamic light scattering

The nanoparticle dimensions can translate into differences observed in solution-based chemistry in, for example, the preparation of polymer composites or emulsion stabilisation.^30,31^ This can affect dispersion organisation into self-assembled chiral nematic liquid crystalline phases.^62^ For example, nanoparticle length and size distribution was attributed to the ability of CNCs to stabilise oil-in-water Pickering emulsions. Smaller, well-defined particles offered greater surface coverage while longer crystallites provided larger steric hindrance that limited denser packing and allowed for porous surfaces and multi-layered organizations.^56^

DLS is often used as a rapid technique to assess the quality and polydispersity of nanoparticle suspensions, and comparisons of light scattering data have been explicitly applied to CNC suspensions.^13,68^ In the case of CNCs, the results are expressed as the apparent hydrodynamic radius (*r*_H_). This is because DLS relies on Brownian motion to monitor scattering intensity. The particle size is determined based on diffusivity *via* the Stokes–Einstein equation;^69,70^ however, this relies on an a constant diffusion coefficient and an assumed spherical hydrodynamic radius. Since the diffusion coefficient perpendicular and parallel to the transverse axis are different, this is not the case for rod-shaped nanoparticles.^71,72^

Examination of the obtained apparent *r*_H_ from the different CNC suspensions (Table 3) indicate no statistical differences between hCNC, pCNC, hCNCi, sCNCi, and pCNCi; however, CNCs obtained from H_2_SO_4_ hydrolysis (sCNC) where smaller and more uniform in size with a lower apparent *r*_H_ (21 ± 8) in agreement with the obtained AFM data. These CNCs also exhibited the largest absolute *ζ*-potential (−42.8 ± 1.3), which is a measure of their colloidal stability determined by electrophoretic light scattering and relates to the surface charge density of the CNCs.

#### Electrophoretic light scattering

Measurements of *ζ*-potential are based upon electrophoretic mobility (*U*_E_) determined using electrophoretic light-scattering.^73^ Generally the Hückel or Smoluchowski approximations of Henry's function are used to calculate the *ζ*-potential from *U*_E_ using Henry's equation. The *U*_E_ depends on the applied electric field and the size of the particles, and for the Henry function assumes a spherical shape. The Hückel and Smoluchowski approximations assume the electric double layer around the particles are, respectively, either much thicker or thinner than the particles themselves. Many of these assumptions tend to breakdown as the particles deviate from spherical shapes.^69,73,74^ Similar effects have been noted previously for applications of other light scattering techniques (*e.g.* DLS) when measuring particle size.

It was previously shown that the amount of surface charge groups (mmol kg^−1^) is strongly correlated with the *ζ*-potential of the CNCs (*r*^2^ ≈ 0.81).^40^ However, the association was not perfect because the surface charge density *σ*, which correlated more strongly with *ζ*-potential (*r*^2^ ≈ 0.87),^40^ is highly dependent on the size of the CNCs as well as differences arising from transverse location and rotation about the axis of particles during measurement. Thus, although a direct standardization of S or P content from *ζ* potential cannot be made, the *ζ*-potential is a useful rapid measure to determine the degree of surface charge and stability of nanoparticle systems.

Generally, CNCs are considered colloidally stable, when the measured absolute zeta(*ζ*)-potential value is >25 mV,^58^ while absolute values between 10–25 mV are considered intermittently stable and may flocculate given enough time or added solute (salt),^75,76^ although neutral solutions may be sterically stabilized.^29^ All CNC samples analysed exhibited a negative *ζ*-potential (Table 3). In the case of CNCs hydrolysed with H_2_SO_4_ or H_3_PO_4_, deprotonation to give either –OPO_3_^2−^ or –OSO_3_^−^ resulted in highly charged CNCs with a rather large negative *ζ*-potential (−20.2 and −42.8, respectively). Despite having a weakly ionisable hydroxyl group (p*K*_a_ of C6 hydroxyl of anhydroglucose ∼12.6), hCNC and hCNCi displayed slightly negative *ζ*-potentials. This is because the concentration of the CNCs tested, given the p*K*_a_ of the hydroxyls at neutral pH, resulted in a slight ionisation of the hydroxyls to give ∼13 μmol kg^−1^ of the resulting alkoxide which were at best intermittently stable. This also accounts for the larger apparent hydrodynamic radius, despite the similarity in absolute size measured by AFM. Samples of sCNCi and pCNCi mediated hydrolysis were at the cusp of colloidal stability with *ζ*-potentials of −24.2 ± 1.7 and −25.0 ± 2.2, respectively.

### X-ray photoemission spectroscopy

X-ray photoemission spectroscopy (XPS) was used for analysis of the nanocrystal elemental composition. In each instance, strong signals were observed at 285 eV and 531 eV, corresponding to C 1s and O 1s, respectively (see ESI, Fig. S4‡). In addition, trace signals were observed at 133 eV or 167 eV, corresponding to the P 2p and S 2p ionisation binding energies in samples containing either phosphate or sulfate half-esters. The summary of results is displayed in Table 3 and compared to the results obtained by conductometric titration. It is shown that for the CNCs with greater functionalisation rates, smaller size, and greater surface area to volume ratio, the value obtained for sCNC by conductometry and XPS are quite similar, 0.84% and 0.83%, respectively. However, for lower functionalisation (sCNCi, pCNC, and pCNCi), a larger amount of S or P was detected in the samples compared to conductometry. There are several contributing factors. First, XPS is a surface sensitive technique that assumes the bulk material is the same as the surface, and second, the lower level of functionalization was near the limit of detection and thus resulted in a lower signal to noise ratio. Finally, the XPS data did not distinguish between residual S or P found in the biomass samples from those introduced as surface acid groups during hydrolysis to produce CNCs.

The XPS results confirm low-level functionalization of the CNCs when treated with either H_2_SO_4_ or H_3_PO_4_. As observed by the conductivity titrations, the surface charge correlates to the number of acidic phosphate esters or sulfate half-esters on the CNC surface. Overall, under the IL-hydrolysis conditions employed, about one-fourth as many anhydroglucose units are functionalised with an –OSO_3_^−^ group compared to the controls utilising standard H_2_SO_4_ hydrolysis conditions. Hydrolysis under harsher conditions with H_3_PO_4_ gave similar values for H_3_PO_4_ IL-hydrolysis.

### Thermogravimetric analysis

**Fig. 3:**
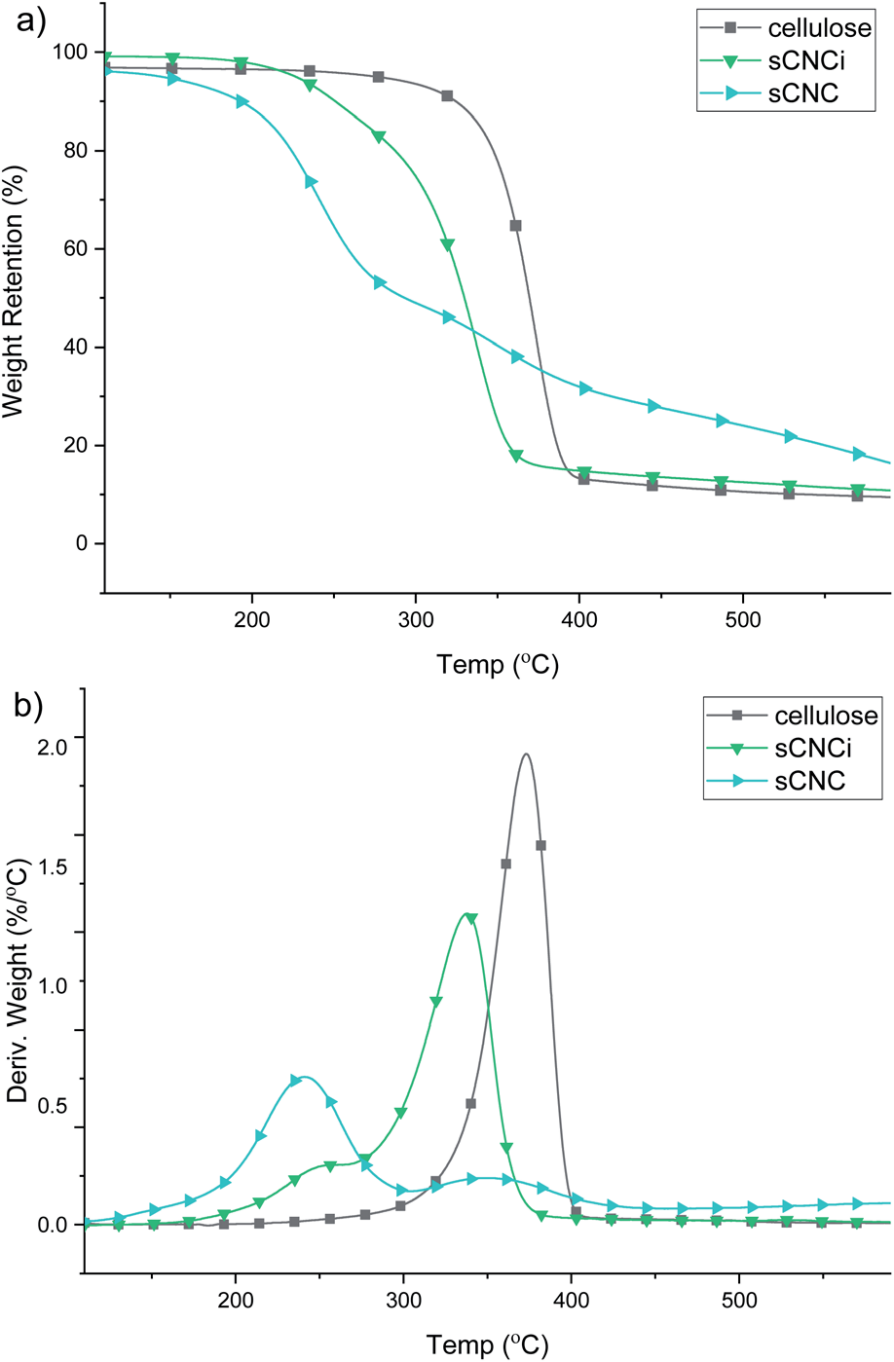
Representative TGA (a) and DTG (b) thermograms for cellulose and sulfate CNCs from traditional (sCNC) and ionic liquid-mediated (sCNCi) hydrolysis of cellulose. Error between individual measurements for each sample was ≤1%.

Analysis of the TGA and first-derivative (DTG) thermograms for native cellulose, compared to the H_2_SO_4_-hydrolysed CNC product (sCNC) and H_2_SO_4_/IL-mediated hydrolysis (sCNCi) is shown in Fig. 3. Samples for hydrolysis using HCl, H_3_PO_4_, and H_2_SO_4_ with and without the IL are shown in the ESI (Fig. S6–S8‡). The TGA thermogram for H_2_SO_4_-hydrolysed CNCs displayed an initial degradation temperature of *ca.* 200 °C corresponding to decomposition of the surface sulfate half-esters, followed by a second DTG local maxima (350 °C) corresponding to the temperature for the decomposition of the cellulose chains.^10,13,77^

Very similar thermal stabilities for hCNC and pCNC are indicated based on the TGA thermograms. The initial onset temperature of thermal degradation (*T*_onset_) for native cellulose was 349 °C and the temperature of the peak maxima (*T*_max_) of the corresponding DTG trace occurred at 374 °C. It has been previously shown that H_3_PO_4_-hydrolysed CNCs are more resistant to thermal decomposition, compared to sCNCs.^10^ One reason for this is that the sulfate half-esters on the CNC surface catalyse the autodegradation of cellulose, especially at higher temperatures,^40,77^ while in contrast phosphorylated CNCs exhibit a much higher thermal stability.^10^ A similar effect was observed in this instance, where the *T*_max_ was 349 °C and the *T*_onset_ was 323 °C for pCNC. HCl-hydrolysed CNCs displayed intermediate behaviour with a single peak *T*_max_ at 342 °C and initial onset temperatures of 292 °C.

By contrast all IL-hydrolysed CNCs display similar thermal decomposition pathways which are likely the result of partial swelling and dissolution of the cellulose chains during the concurrent IL treatment. This has been linked to a reduction in crystallinity and hence thermal stability of the CNCs. Additionally, IL-hydrolysed CNCs possess a similar abundance of surface charged groups, which do not significantly affect their thermal properties at low functionalization rates. As such, only modest differences were observed; pCNCi from IL-mediated hydrolysis were slightly more stable than sCNCi or hCNCi. The *T*_onset_ for IL-hydrolysed sCNCi, hCNCi, and pCNCi was 297, 298, and 308 °C, respectively, while the *T*_max_ was 342, 342, and 356 °C.

### Crystallinity

**Fig. 4:**
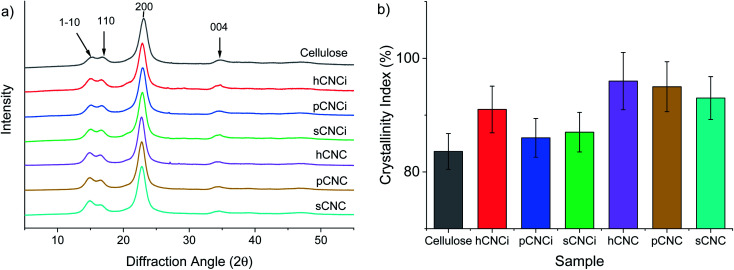
XRD spectra for cellulose and nanocellulose from inorganic acid and IL-mediated hydrolysis of cotton gin motes (a); and, calculated crystallinity index using the MAUD Rietveld refinement program (b). The error bars represent estimated uncertainty in the fit of the calculated diffraction pattern.

The crystallinity of CNCs has been linked to a toughening effect in polymer composites^32^ and some pre-treatments, including swelling and hydrolysis methods (*e.g.* mercerization), are known to affect cellulose crystallinity and/or convert cellulose I to cellulose II,^15,42,46,78^ which can ultimately affect their solubility, thermal stability, and other physical properties.^62,78,79^ Therefore, it is important to ascertain the crystalline state of CNCs and the crystallinity index (CrI) when considering potential applications. Samples were analysed for CrI using powder X-ray diffraction (XRD). The MAUD Rietveld refinement program was used for analysis of the resulting diffraction patterns (see ESI Fig. S9‡).^46,80^ The diffraction patterns for native cellulose, the prepared CNCs and IL-CNCs are shown in Fig. 4a. The predominant reflections observed for the various CNC derivatives and those from IL hydrolysis were of the (1−10), (110), (200) and (004) peaks corresponding to the cellulose 1β diffraction pattern located at approximately 14.8°, 16.5°, 22.8°, and 34.7° 2*θ*, respectively.

The crystallinity (Fig. 4b) and crystallite size were determined using eqn (4) and (5), respectively, while the *d*-spacings were calculated from the refined unit cell dimensions. CNCs prepared using IL-mediated hydrolysis were highly crystalline with an average crystallinity index of 88%; this is only slightly reduced compared to CNCs produced from mineral acid hydrolysis (95%) and considerably higher than CNCs produced using neat IL from some wood sources^26,27^ and on par with those reported from cotton linters or cotton pulp.^16^ This is likely due to the relatively high crystallinity of the native cellulose used for the preparation of CNCs and mild conditions employed during IL-hydrolysis. Harsher conditions, or prolonged swelling prior to acid hydrolysis have previously been shown to severely alter cellulose crystallinity.^17,81^ Specifically, swelling and dissolution in ILs has been shown to disrupt the crystalline regions of the cellulose chains increasing the amorphous content,^82,83^ this partially explains the changes in the TGA and DTG thermograms (Fig. S6 and S8‡) as well as the slight reduction in crystallinity observed (Fig. 4b) compared to CNCs from traditional methods.

**Fig. 5:**
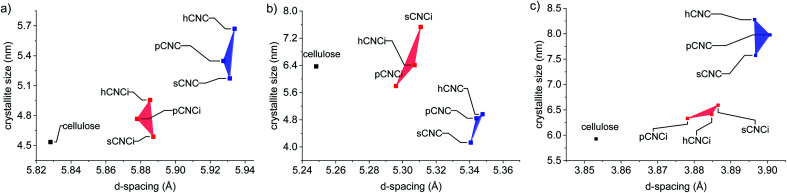
Relationship between *d*-spacing and crystallite size of the lattice planes: (a) (1−10); (b) (110), and; (c) (200).

To further ascertain variations in the crystalline structure as the result of the IL treatment, the crystallite sizes and *d*-spacings were plotted for the main lattice plains of cotton cellulose (Fig. 5). Compared to native cellulose mineral acid hydrolysis results in increased *d*-spacing along the (1−10) lattice plane and accordingly an increase in crystallite size along that dimension. This correlated with a decrease in crystallite size along the (110) lattice plane. Under the milder conditions employing the IL, the calculated *d*-spacings and crystallite sizes were more akin to native MCC than to CNCs produced from mineral acid hydrolysis. Thus, the dilute IL and dilute acid conditions did less to perturb the native cellulose structure than concentrated acid hydrolysis.

## Conclusions

This work represents a facile strategy to obtain cellulose nanocrystals using dilute acids in combination with ionic liquids under mild conditions. A single ionic liquid ([BMIm]Cl) was used for the extraction of nanocellulose using three inorganic acids, showing the generality of these conditions. CNCs obtained *via* this method are larger than CNCs obtained by conventional methods, but with similar aspect ratio, thus, maintaining favourable properties such as high crystallinity and similar crystallite sizes. Moreover, IL-mediated hydrolysis improves the thermal properties of sulfate CNCs, potentially expanding their applications in melt extrusion and polymer composites. Additionally, IL-hydrolysed CNCs with longer aspect ratios may prove beneficial for preparation of gas adsorbents and for emulsion stabilisation. Future work will focus on optimizing the reaction conditions, the selection of the specific IL in combination with different acids to improve yield and CNC recovery and employing IL-hydrolysed CNCs in suitable material applications.

## Conflicts of interest

There are no conflicts to declare.

## Supplementary Material

RA-010-D0RA05976E-s001
