# Supplementary material for: Cellulose hydrolysis using ionic liquids and inorganic acids under dilute conditions: morphological comparison of nanocellulose
Source: RSC Adv. 2020 Oct 28;10(65):39413–24. doi: 10.1039/d0ra05976e (PMC9057393; doi:10.1039/d0ra05976e)
Supplement: RA-010-D0RA05976E-s001 [file RA-010-D0RA05976E-s001.pdf]

Electronic Supplementary Material (ESI) for RSC Advances.
This journal is © The Royal Society of Chemistry 2020

RSC Advances

Supplementary Information

Cellulose hydrolysis using ionic liquids and inorganic acids under dilute conditions: Morphological comparison of nanocellulose

Jacobs H. Jordan, Michael W. Easson,* and Brian D. Condon

USDA, Agricultural Research Service, Southern Regional Research Center, 1100 Robert E. Lee Blvd, New Orleans, LA 70124, USA

*To whom correspondence should be addressed. Email: Michael.easson@usda.gov

Jacobs H. Jordan https://orcid.org/0000-0002-0238-3864
Michael W. Easson https://orcid.org/0000-0002-2268-1922
Brian D. Condon https://orcid.org/0000-0003-1944-021X

List of Figures, Tables, & Schemes

Scheme S1 Extraction of cellulose from cotton gin motes using mechanical and chemical treatments. 2

Table S1. Volume, surface area, total area, amount of surface charged groups and charge density from conductivity experiments............ 2
Table S2. Elemental composition of CNCs from XPS 5

Fig. S1 Conductivity curves averaged over three titrations for the different CNCs. Notably, only sCNC contain a significant proportion of surface strong acid groups 2
Fig. S2 AFM length and height analysis of CNCs prepared by mineral acid hydrolysis. a) hCNC length histogram; b) hCNC height histogram; c) sCNC length histogram; d) sCNC height histogram; e) pCNC length histogram; f) hCNC height histogram 3
Fig. S3 AFM length and height analysis of CNCs prepared using dilute acids and ionic liquid mediated hydrolysis. a) hCNCi length histogram; b) hCNCi height histogram; c) sCNCi length histogram; d) sCNCi height histogram; e) pCNCi length histogram; f) hCNCi height histogram 4
Fig. S4 XPS survey scans of the different CNCs produced............ 5
Fig. S5 2% (w/w) suspensions of IL-prepared CNCs at t = 0 d and after 90 d 5
Fig. S6 Representative TGA (a) and DTG (b) thermograms for cellulose, hCNC, and hCNCi............ 6
Fig. S7 Representative TGA (a) and DTG (b) thermograms for cellulose, sCNC, and sCNCi............ 6
Fig. S8 Representative TGA (a) and DTG (b) thermograms for cellulose, pCNC, and pCNCi............ 6
Fig. S9 MAUD Rietveld refinements for CNCs from mineral acid hydrolysis and dilute acid/IL-mediated hydrolysis 7

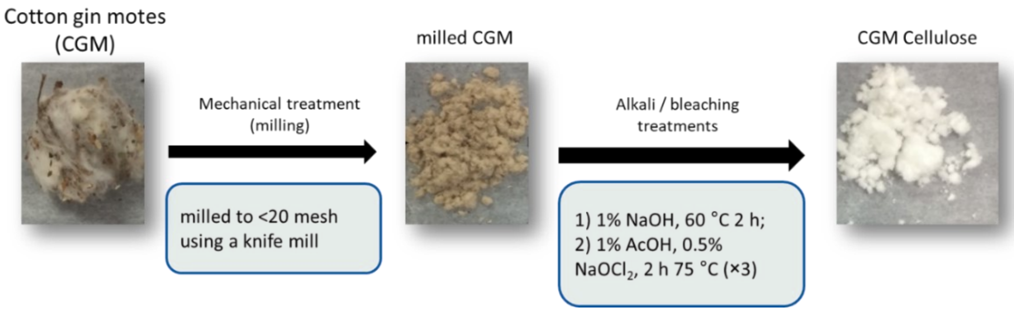

Scheme S1 Extraction of cellulose from cotton gin motes using mechanical and chemical treatments

The amount of surface charge groups (mmol·kg^{-1}) can be determined directly from conductometric titration (**Fig. S1**)[1, 2]

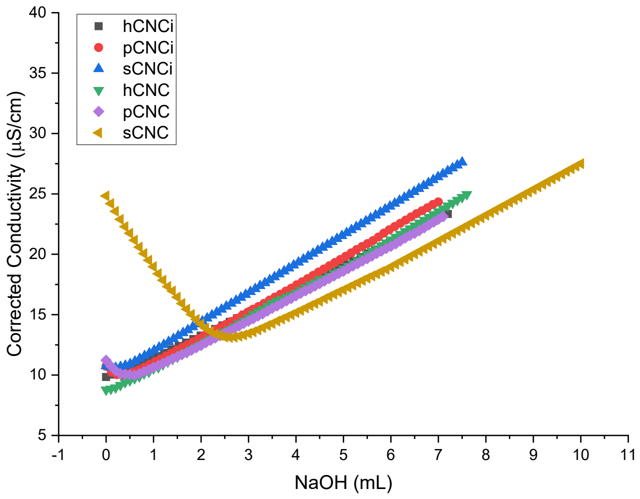

Fig. S1 Conductivity curves averaged over three titrations for the different CNCs. Notably, only sCNC contain a significant proportion of surface strong acid groups

The surface charge density in e/nm2 can be calculated from the amount of S or P determined from conductometric titrations (**Fig. S1**).[3] The surface charge density σ is determined from the total mole equivalents (mol) of S or P in 1 g of CNCs by Eq. (S1):[3]

$$\sigma = -e\,\frac{mol \cdot N_A}{A_{total}} \quad (S1)$$

Where e is the elementary charge, N_A is Avogadro's number, and A_{total} is the surface area of the CNCs calculated from Eq. (S2):

$$A_{total} = \frac{A_{rod}}{\rho\, V_{rod}} \quad (S2)$$

Where ρ is the density of cellulose (1.6 g/cm^3),[3] A_{rod} is the average surface area of a rod-shaped nanocrystal, and V_{rod} is the average volume of a rod-shaped nanocrystal. For simplicity, the CNCs in this case were assumed to be cylindrical, and calculations were based on the average length and height determined by AFM (**Fig. S2** and **Fig. S3**).

Table S1. Volume, surface area, total area, amount of surface charged groups and charge density from conductivity experiments

Sample	Vol[a] (nm^3)	Surface Area [a] (nm^2)	Total Area [b] (nm^2)	S or P [c] (mmol·kg^{-1})	σ [d] (e/nm^2)	% S or % P
**sCNC**	4153	2307	3.59×10^{20}	260 ± 6	-0.436 ± 0.010	0.83%
**pCNC**	9543	5178	3.39×10^{20}	36 ± 1	-0.064 ± 0.002	0.11%
**sCNCi**	14632	6182	2.64×10^{20}	20 ± 7	-0.046 ± 0.016	0.06%
**pCNCi**	32867	11736	2.23×10^{20}	34 ± 4	-0.092 ± 0.011	0.11%

a) per nanocrystal; determined assuming a cylindrically shaped rod; b) per gram of cellulose nanocrystals calculated using Eq. S2; c) determined from conductometric titrations; d) calculated from S or P content using Eq. S1

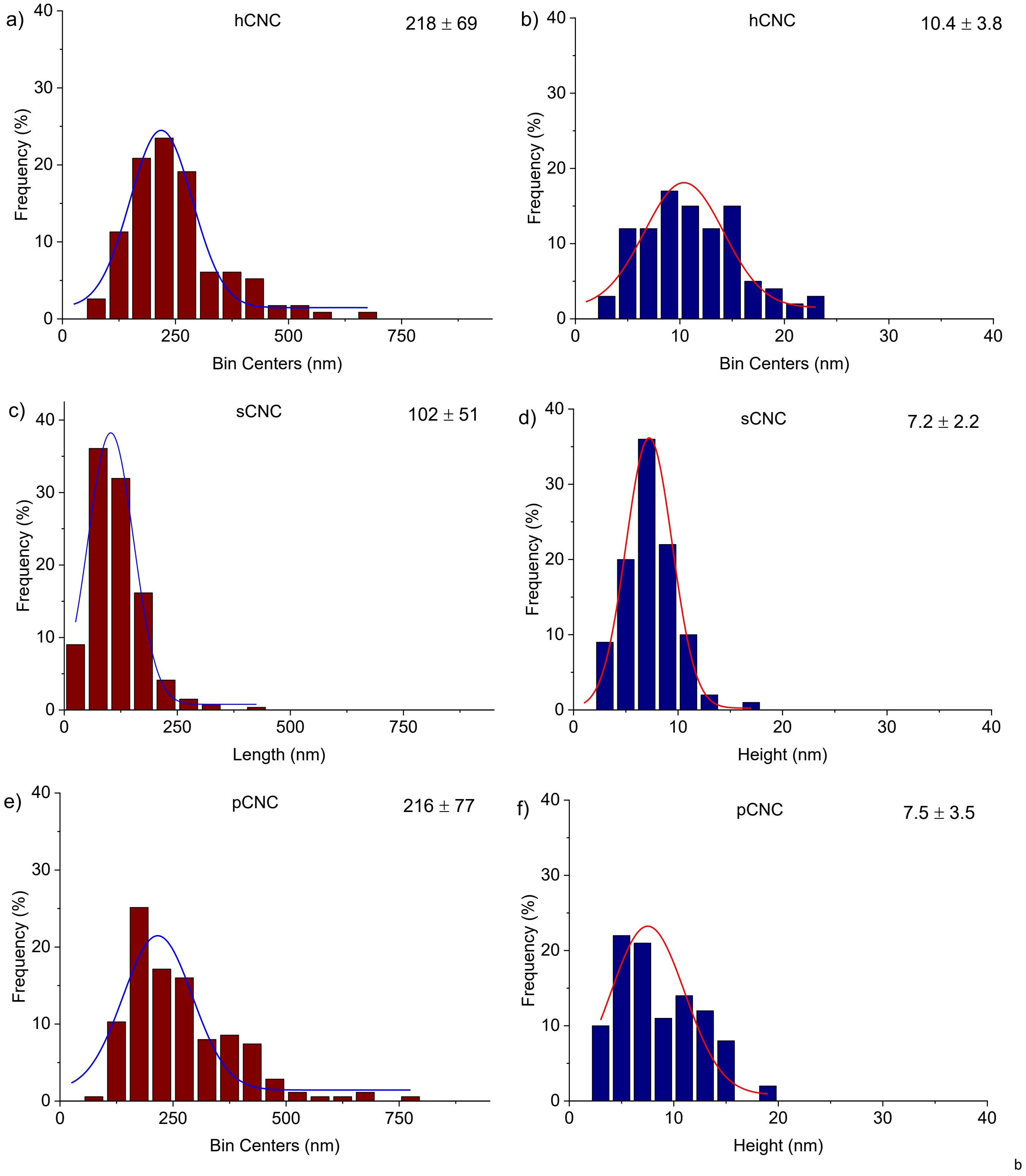

Fig. S2 AFM length and height analysis of CNCs prepared by mineral acid hydrolysis. a) hCNC length histogram; b) hCNC height histogram; c) sCNC length histogram; d) sCNC height histogram; e) pCNC length histogram; f) hCNC height histogram

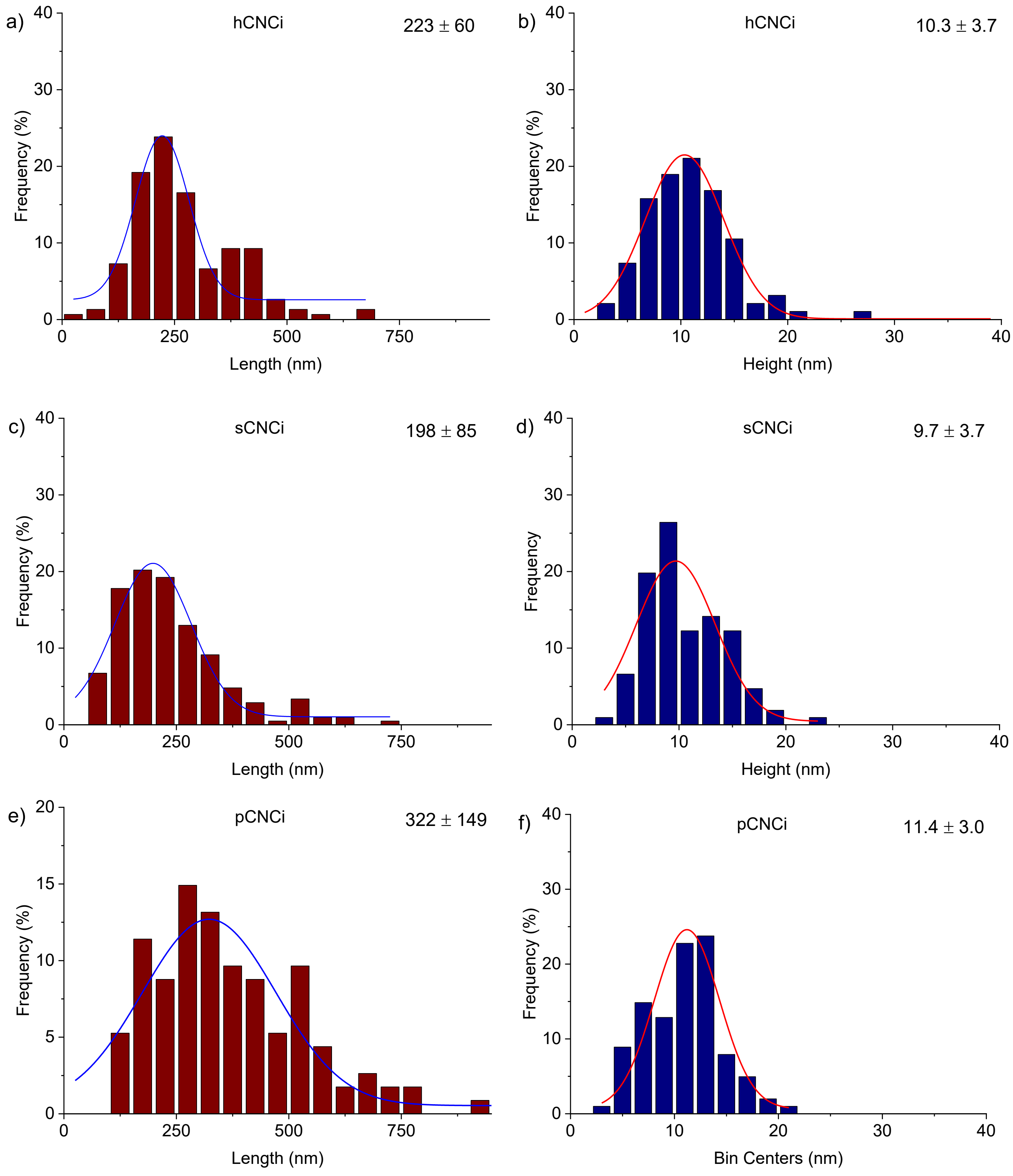

Fig. S3 AFM length and height analysis of CNCs prepared using dilute acids and ionic liquid mediated hydrolysis. a) hCNCi length histogram; b) hCNCi height histogram; c) sCNCi length histogram; d) sCNCi height histogram; e) pCNCi length histogram; f) hCNCi height histogram

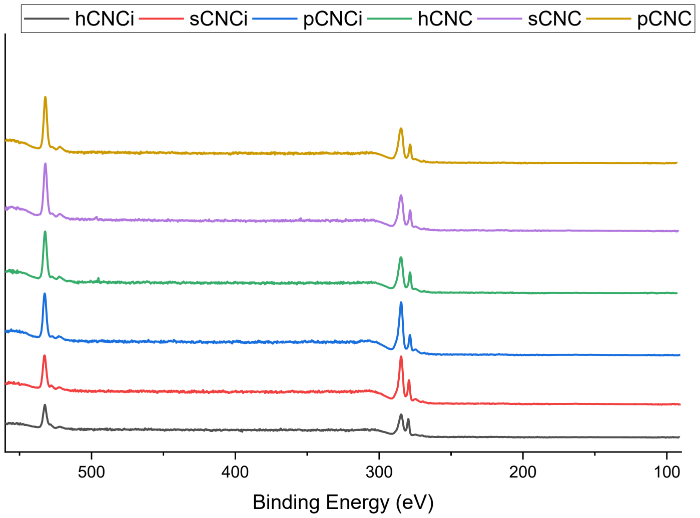

Fig. S4 XPS survey scans of the different CNCs produced

Table S2. Elemental composition of CNCs from XPS

Entry	Sample	C [a] (%)	O [a] (%)	S [a] (%)	P [a] (%)
**1**	hCNC	42.50	57.50	0.00	0.00
**2**	sCNC	39.26	59.90	0.84	0.00
**3**	pCNC	41.88	57.87	0.00	0.25
**4**	hCNCi	50.70	49.30	0.00	0.00
**5**	sCNCi	51.17	48.63	0.20	0.00
**6**	pCNCi	50.40	49.38	0.00	0.22

[a] Elemental mass % calculated from atom % from XPS

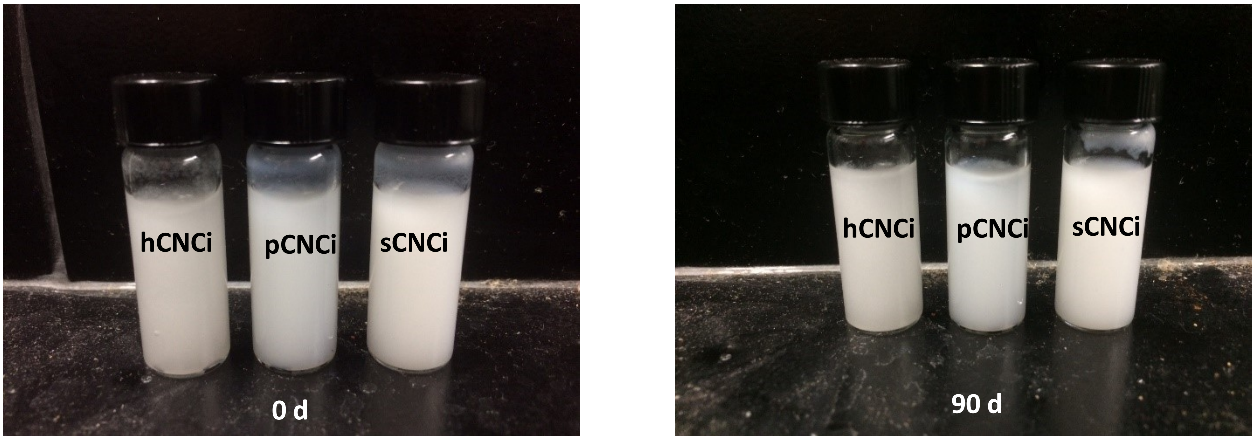

Fig. S5 2% (w/w) suspensions of IL-prepared CNCs at t = 0 d and after 90 d

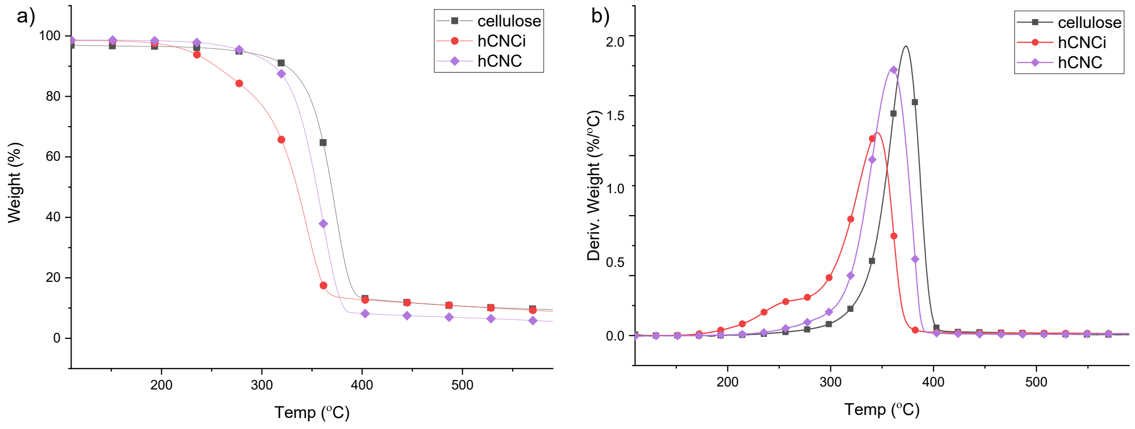

Fig. S6 Representative TGA (a) and DTG (b) thermograms for cellulose, hCNC, and hCNCi.

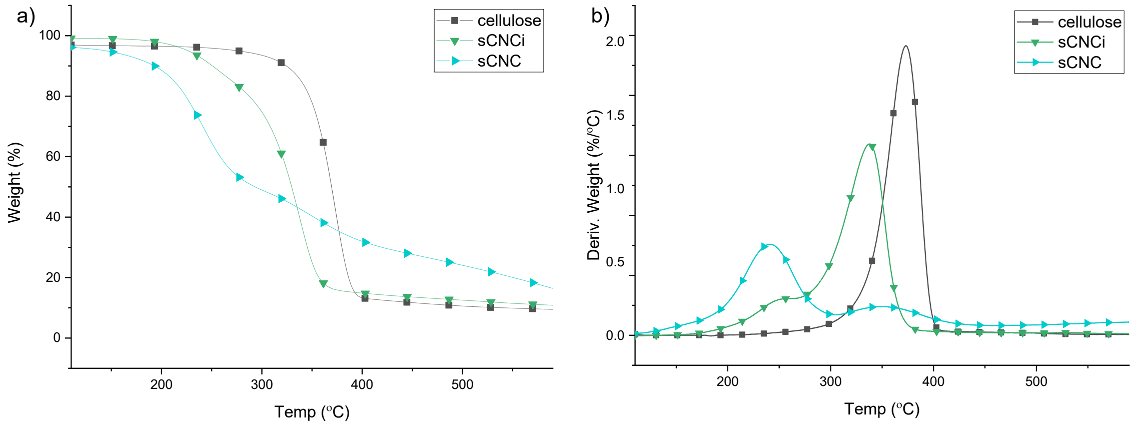

Fig. S7 Representative TGA (a) and DTG (b) thermograms for cellulose, sCNC, and sCNCi.

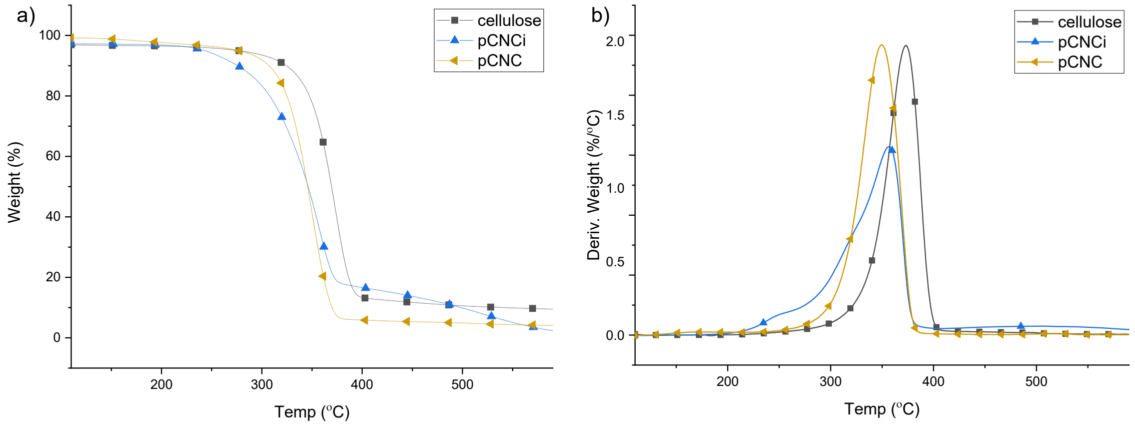

Fig. S8 Representative TGA (a) and DTG (b) thermograms for cellulose, pCNC, and pCNCi.

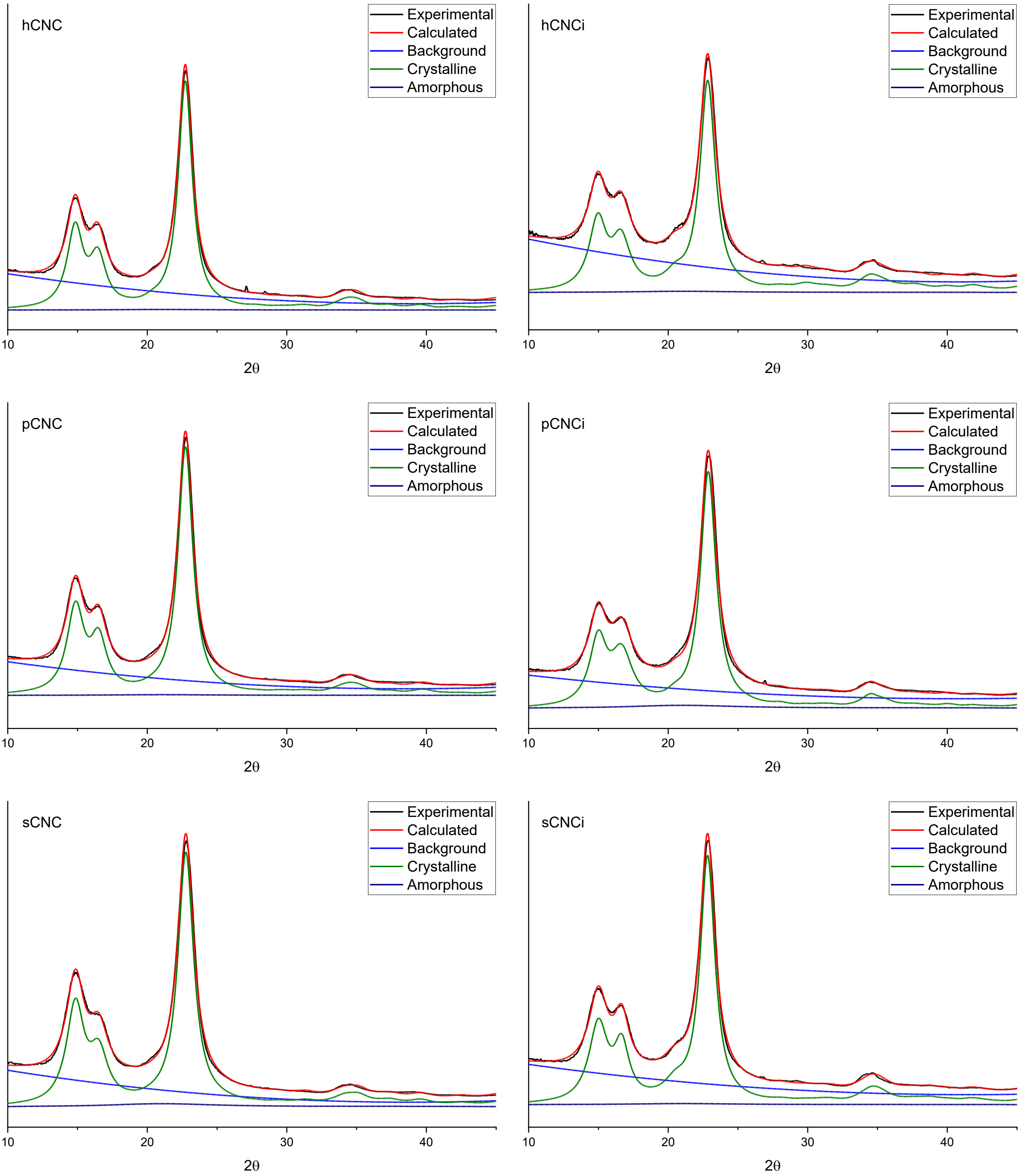

Fig. S9 MAUD Rietveld refinements for CNCs from mineral acid hydrolysis and dilute acid/IL-mediated hydrolysis

References

1. S. Beck, M. Méthot and J. Bouchard, *Cellulose*, 2014, **22**, 101-116.
2. J. H. Jordan, M. W. Easson and B. D. Condon, *Nanomaterials*, 2019, **9**, 1232.
3. F. Jiang, A. R. Esker and M. Roman, *Langmuir : the ACS journal of surfaces and colloids*, 2010, **26**, 17919-17925.
